# Nurturing families: A feasibility randomised controlled trial of a whole-family intervention with vulnerable families in Jordan

**DOI:** 10.1017/gmh.2024.43

**Published:** 2024-04-11

**Authors:** Felicity L. Brown, Hind Yousef, Alexandra C.E. Bleile, Hadeel Mansour, Anna Barrett, Maha Ghatasheh, Eve S. Puffer, Zeinab Mansour, Karam Hayef, Samer Kurdi, Qaasim Ali, Wietse A. Tol, Aala El-Khani, Rachel Calam, Hana Abu Hassan, Mark J.D. Jordans

**Affiliations:** 1 Research and Development Department, War Child Alliance, Amsterdam, The Netherlands; 2 Research and Development Department, War Child Alliance, Amman, Jordan; 3 Amsterdam Institute of Social Science Research, University of Amsterdam, Amsterdam, The Netherlands; 4 Department of Psychology and Neuroscience, Duke Global Health Institute, Duke University, Durham, NC, USA; 5 Collateral Repair Project, Amman, Jordan; 6 Section of Global Health, University of Copenhagen, Copenhagen, Denmark; 7 Athena Research Institute, VU University Amsterdam, Amsterdam, The Netherlands; 8 Arq International, Diemen, The Netherlands; 9 Division of Psychology and Mental Health, The University of Manchester, Manchester, UK; 10 University of California San Diego, San Diego, CA, USA; 11 Imperial College NHS Trust, London, UK

**Keywords:** family interventions, mental health, Jordan, refugee, pilot randomised controlled trial

## Abstract

Armed conflict and forced displacement can significantly strain nurturing family environments, which are essential for child well-being. Yet, limited evidence exists on the effectiveness of family-systemic interventions in these contexts. We conducted a two-arm, single-masked, feasibility Randomised Controlled Trial (fRCT) of a whole-family intervention with Syrian, Iraqi and Jordanian families in Jordan. We aimed to determine the feasibility of intervention and study procedures to inform a fully-powered RCT. Eligible families were randomised to receive the Nurturing Families intervention or enhanced usual care (1:1). Masked assessors measured outcomes at baseline and endline; primary outcome measures were caregiver psychological distress, family functioning, and parenting practices. Families and implementing staff participated in qualitative interviews at endline. Of the 62 families screened, 60 (98%) were eligible, 97% completed the baseline and 90% completed the endline. Qualitative feedback indicated specific improvements in adolescent well-being, caregiver distress and parenting, and family relationships. Data highlighted high participant engagement and adequate facilitator fidelity and competence. Outcome measures had good psychometric properties (most α > 0.80) and sensitivity to change, with significant changes seen on most measures in the intervention but not control group. Findings indicate the acceptability and feasibility of intervention and study procedures. Subsequent full-scale evaluation is needed to determine effectiveness.

## Impact statement

Armed conflict and forced displacement can significantly disrupt family functioning, leading to strains across the family system. In this feasibility RCT, we demonstrate the feasibility of taking a whole-family approach to mental health and psychosocial support in refugee settings and providing holistic care for families facing multiple psychosocial challenges. Our findings indicate the viability of delivery through non-specialist community-based facilitators, which has the potential to increase scalability and significantly close the large treatment gap in such settings. Based on the results of this study, we recommend that a full-scale evaluation of the Nurturing Families intervention be conducted to determine effectiveness.

## Background

Nurturing family environments are essential for child and adolescent development, mental health and well-being (Biglan et al., 2012). Yet, when families face significant adversities, including armed conflict and forced displacement, they experience increased daily stressors (Miller and Rasmussen, 2010), heightened risk of psychological distress and disorders (Charlson et al., 2019) and strains in family relationships (Barrett et al., under review), all of which can negatively impact child well-being. Increases in armed conflicts and other humanitarian emergencies have led to the current record level of displaced individuals globally, yet the majority live in low- and middle-income countries (LMIC; UNHCR, 2023a) with under-resourced health and social protection systems (WHO, 2021). This commonly results in high levels of mental health needs but limited available services (Evans-Lacko et al., 2018).

There is growing evidence that empirically-supported intervention techniques can be successfully manualised and culturally and contextually adapted to different conflict-affected settings (Barbui et al., 2020). This includes delivery by trained and supervised lay-people through a ‘task-sharing’ approach, allowing greater scalability through increasing the available workforce and enhancing local fit (Cohen and Yaeger, 2021). However, most existing intervention research and practice focuses on approaches that address individual-level stressors and coping (Barbui et al., 2020). Although important, individual-level interventions often fail to address the complex influences on child and adolescent mental health across different levels of the social ecology. Caregivers and families affected by armed conflict are exposed to severe and prolonged stress and adversity, often against a backdrop of structural inequity and poverty, and may struggle to provide responsive parenting, which has a significant impact on family dynamics and subsequent child outcomes (Barrett et al., under review; Eltanamly et al., 2021; Panter-Brick et al., 2014; Sim et al., 2018). Influences within the family system therefore act as powerful risk or protective factors – further compounding or mitigating impacts of conflict and forced displacement on children. There is an emergent literature on promising approaches that work with the entire family or multiple family members (‘whole family’; e.g., Betancourt et al., 2020; El-Khani et al., 2022; Puffer et al., 2020, 2021), and some pilots of whole-family skills-building interventions for families in humanitarian settings (e.g., Puffer et al., 2017; Haar et al., 2020), but to date there have been no fully-powered randomised controlled trials of interventions evaluated for families that are facing significant distress in humanitarian settings (Bosqui et al., 2024; Pedersen et al., 2019).

To address this gap, we developed a new whole-family intervention targeting family-system interactions (‘family-systemic’), drawing on evidence-based intervention strategies, and developed through a collaborative process with affected communities (Brown et al., under review). The Nurturing Families (NF) intervention builds on an existing brief single-module family-systemic intervention developed in Lebanon for adolescents with heightened emotional distress (Brown et al., 2022). Recognising that families commonly face multiple psychosocial challenges, NF is a modular intervention aiming to provide holistic care for multiple psychosocial challenges, including family interactions, caregiver mental health and well-being, and parenting support (Brown et al., in preparation), with the assumption that improvements in these family domains will impact child and adolescent mental health and well-being. It applies task-sharing principles to delivering whole-family support, which have previously shown effectiveness for adult distress (Bryant et al., 2022), adolescent distress (Bryant et al., 2022), parenting (Puffer et al., 2015) and caregiver mental health (Miller et al., 2022). A small case series study (Brown et al., under review) showed feasibility, relevance, and acceptability of delivering the intervention.

Following recommendations for developing complex interventions (Skivington et al., 2021), we next conducted this feasibility randomised controlled trial (fRCT) with 60 families to assess the feasibility of intervention and study procedures and inform necessary adaptations prior to a fully-powered RCT. Our primary hypotheses were i) outreach, screening, attendance and retention rates for NF intervention and endline assessments will be high and indicate the feasibility of a full RCT; ii) the intervention will be feasible, relevant and acceptable. Additional hypotheses were iii) outcome measures will show sound psychometric properties, including sensitivity to change, with trends in improvement over time in the intervention group but not the control group; iv) trial procedures (randomisation, masking, safety monitoring, spill-over) will be feasible, safe, and acceptable to families.

## Methods

### Design

Between March and July 2022 we conducted a single-masked, two-arm fRCT randomly allocating families (1:1) to Intervention or Enhanced Usual Care (EUC) with an embedded qualitative process evaluation. We assessed: i) outreach, screening, attendance, and retention; ii) fidelity and competence of facilitators; iii) feasibility of randomisation and masking, and spill-over between groups; iv) psychometric properties and trends in outcome measures from baseline to endline (see Table 1). The study was registered retrospectively (ISRCTN76902687, protocol available on request), and is reported following CONSORT guidelines (Eldridge et al., 2016; see Supplementary material). Ethical approval was obtained from Jordan University of Science and Technology (#80/147/2022; 21/02/2022).

**Table 1.** Nurturing Families feasibility RCT hypotheses, data collected, and findings

Study hypothesis	Data collected	Indicator	Findings
i) Outreach, screening, attendance, and retention rates for NF intervention and endline assessments will be high and indicate feasibility of a full RCT	Outreach experiences	High interest is demonstrated in community based on high attendance at screening assessments	Supported
	Screening rates	Screening process results in sufficient rates of inclusion	Supported
	Attendance in sessions	High rates of attendance in NF sessions	Supported
		Equivalent attendance between groups in EUC and other services	Supported
	Retention rates at endline assessments	High completion of endline assessments (>80%)	Supported
	Qualitative facilitators and barriers to engagement	Qualitative findings indicate feasibility of uptake and engagement	*Partially—some barriers identified*
ii) The intervention will be feasible, relevant, and acceptable	Participant perceptions of feasibility, relevance, and acceptability of intervention	Qualitative findings indicate high perceptions of feasibility, relevance, and acceptability	Supported
	Facilitator fidelity – facilitator–report	Facilitators implement intervention with high fidelity ratings on session checklists	Supported
	Facilitator fidelity – observer–report		
	Facilitator competency– core competencies	Facilitators implement intervention with competence	Supported
	Facilitator competency– intervention–specific		
	Participant experiences of facilitators	Delivery by non–specialists perceived as acceptable	Supported
iii) Outcome measures will show sound psychometric properties, including sensitivity to change with trends in improvement over time in NF but not EUC	Psychometric properties of measures	High cronbach’s alpha	Supported
	Sensitivity to change	Significant changes seen in NF group but not EUC group	*Partially—some adolescent outcomes showed no change*
	Participant perceptions of impact	Qualitative findings indicate perceived positive impacts	Supported
iv) Trial procedures (randomisation, masking, safety monitoring, spill–over) will be feasible, safe, and acceptable	Randomisation results	Procedure results in equivalent groups	Supported
		Randomisation is acceptable to participants	*Not supported—some dissatisfaction*
	Masking	Assessors remain masked	*Not supported—masking not maintained*
	Spill–over	Intervention content is not shared with control group	*Partially—some participants report sharing*
	Safety–monitoring	No adverse events attributable to intervention or study	Supported

### Setting

Jordan hosts approximately 740,000 refugees registered with the United Nations High Commission for Refugees (primarily from conflicts in Syria and Iraq), and 2.4 million Palestinian refugees registered with the United Nations Relief and Works Agency for Palestinian Refugees in the Near East (UNHCR, 2023b), in a total population of approximately 11 million (United Nations, 2022). Refugee populations in Jordan largely live outside of formal camp settings and have reported high levels of psychological distress, exacerbated by multiple environmental stressors, including: insecure income and housing, child labour, restricted access to essential services, and structural and community discrimination (Wells et al., 2016). We conducted this study in a community centre in Hashmi al Shamali – an urban area in Amman characterised by social and economic disadvantages.

### Participants and sample size

We enrolled families of any nationality (the obtained sample was Iraqi, Syrian and Jordanian) meeting the following inclusion criteria: (i) had an adolescent aged 10–17 years; (ii) caregivers and adolescents provided consent/assent; (iii) screened positive for two or more psychosocial problems based on self-report measures (adolescent or caregiver psychological distress, parenting or family functioning challenges); iv) all family members reported no concerns or risks in taking part as a family unit. Given the high rates of adversity for all families in the neighbourhood, and the imperative of humanitarian aid to also support host communities, Jordanian families were included in the study, as well as those with refugee backgrounds. Exclusion criteria were (i) no legal adult caregiver able to provide consent, (ii) significant cognitive or neurological impairments that would prevent participation in intervention or assessment; (iii) imminent risk of suicide or other urgent mental health or protection needs necessitating specialist services. We aimed to enrol 30 families in each arm as this was considered sufficient to answer research questions centred on feasibility in line with previous similar studies in the region (e.g., Brown et al., 2023).

### Outreach, eligibility, consent, and screening

To reach eligible families, we created an adapted family version of the ReachNow outreach tool (van den Broek et al., 2023), a proactive case detection tool that uses illustrated vignettes and a simple decision algorithm to identify children in need of mental health care in community settings. Trained community members used the tool to identify families, introduced the intervention using a structured script, and shared contact details with the study team if the family was interested to take part. The study team then obtained informed consent from the family and conducted a structured screening interview assessing: psychological distress in caregivers (Kessler-10 (K10); cut-off ≥20 (Kessler et al., 2003); caregiver-report of emotional and behavioural problems for adolescents 10–17 years (Paediatric Symptom Checklist-35 [PSC-35]; cut-off ≥21) (Jellinek et al., 1999); caregiver-reported parenting and family functioning challenges; and a single question assessing perceived risk related to attending whole-family sessions. Eligible families immediately completed caregiver baseline assessments, and adolescents were invited to a separate baseline interview. Written informed consent from caregivers was obtained prior to screening, and assent from adolescents was obtained prior to baseline. Verbal assent was taken again prior to endline.

### Interventions

#### Nurturing Families

**Figure 1.**
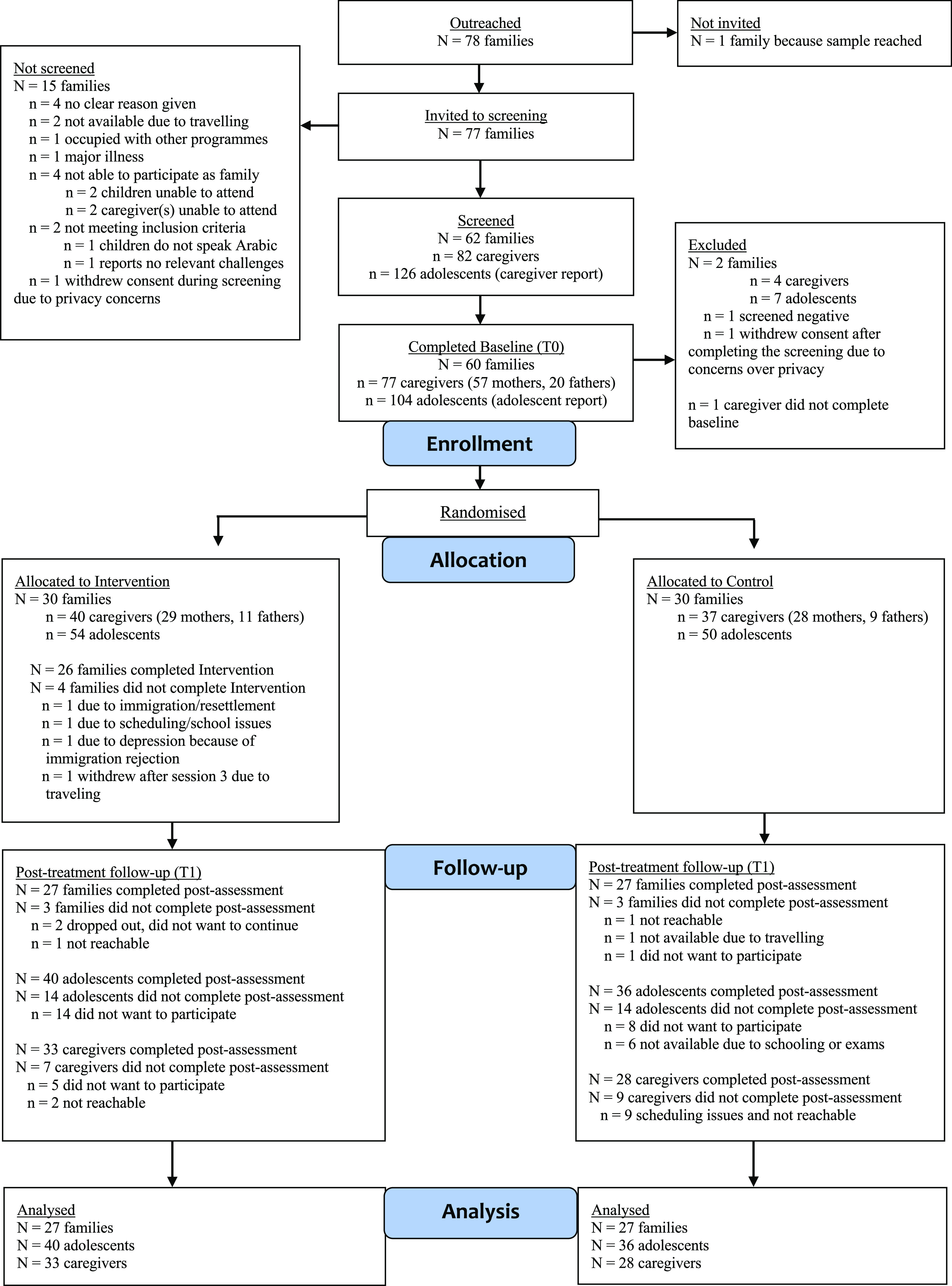
The Nurturing Families intervention outline.

NF is a modular, multi-component, family-systemic approach providing holistic and integrated support to families in managing multiple psychosocial challenges, targeted towards their specific needs. Overall, it aims to improve caregiver and child mental health through strengthening supportive family interactions (Brown et al., under review). The core module contains six 90-min whole-family sessions delivered weekly, followed immediately by brief 30-min check-ins with caregivers. Components were drawn from existing evidence (Bosqui et al., under review), qualitative research and participatory development workshops, and include developing a joint understanding of the family’s strengths, challenges, values and goals; grounding techniques to improve emotional regulation; strategies to improve family communication and perspective taking; joint problem-solving strategies; and conflict management strategies (see Figure 1 for an outline of the intervention). In a subsequent ‘transition’ session, families review progress and future goals and jointly decide which optional advanced modules to follow and for how many sessions, depending on their identified needs. These modules cover: i) solving disagreements (1-2 sessions, whole family); ii)strengthening parenting (2-4 sessions, caregivers only); iii) managing difficult thoughts and feelings (2-4 sessions, caregivers only). Handouts and audio-recordings summarising key content and home practice tasks are provided to facilitate home practice, and to allow sharing of content with non-attending family members.

#### Enhanced usual care

Usual care for families living in Hashmi Al Shamali usually consists of very limited mental health services. Therefore, to ensure an ethical response to vulnerable families identified as having multiple psychosocial challenges, all families (both treatment and control condition) received EUC. This involved i) receiving a list of services available in the community, ii) referral of urgent needs to case management and iii) invitation to a financial literacy course consisting of three, three-hour group sessions.

### Facilitators

Five non-specialist facilitators (two males, three females; without specialist mental health training) delivered the intervention, with two volunteers supporting implementation. They were recruited through the community centre’s networks and selected based on past experience conducting mental health and psychosocial activities and working with children, adults, and families in the community. Training was conducted by an experienced local trainer (a social worker) and consisted of 16 staggered classroom-based days following a structured curriculum introducing core and advanced module content, competencies for working with families, suicide risk assessments and safety planning, safe identification and referral, child protection and safeguarding, and extensive role-plays. Facilitators subsequently implemented the intervention with 12 families under close supervision, followed by a refresher training prior to this study. Weekly group supervision was provided, and the trainer/supervisor received regular supervision from a Jordanian psychologist and an Australian psychologist. Early piloting indicated that the gender of the facilitator did not systematically impact family satisfaction.

### Attendance, Fidelity, and competency

We measured the attendance of individual caregivers and adolescents in intervention and EUC sessions. Facilitators completed session checklists for each session as a measure of facilitator-reported intervention fidelity. The trainer also observed 10% of sessions and rated: i) session components delivered (intervention fidelity; scored as % of components delivered); ii) how well each component was delivered (intervention-specific competency; scored on a three-point scale: ‘done well’, ‘partly done’, ‘needs improvement’) and iii) facilitators’ demonstration of core-competencies (Jordans et al., 2021) and three additional competencies specific to family-level interventions. The observer attended to specific facilitator behaviours for each competency, classified as ‘unhelpful or potentially harmful’, ‘basic helping skills’, and ‘advanced helping skills’.

### Outcome assessments

Baseline was conducted no more than 3 weeks before intervention, and endline within 1 month of the final session (average 16.29 days, range 0–29). Trained and supervised assessors conducted face-to-face interview assessments using Kobo software on tablets. Participants received reimbursement for transportation costs to attend the assessments (5 Jordanian Dinar [JD], approximately 7 USD, per family). Where participants did not attend an assessment, multiple rescheduling attempts were made.

Outcome measures are outlined in Table 2 and were selected based on psychometric properties and appropriateness for the setting, determined in consultation with local study advisors. A rigorous translation process included forward and back-translations by independent bilingual team members, translation workshops, and cognitive interviewing. Demographic data were collected at baseline from caregivers. At endline, caregivers were asked which other services their families accessed.

**Table 2.** Outcome measures used in Nurturing Families feasibility RCT

Domain	Construct	Outcome measure	Added items*	Number of items (total score range)	Improvement indicated by score increase or decrease?
Caregiver	Psychological distress	Kessler 10 (K10; Kessler et al., 2003	None	10 (0–60)	Decrease
	Parenting practices (warmth and responsiveness, positive parenting, harsh punishment)	Arabic Dimension of Parenting Scale (Miller et al., 2022)	1 item on parental problem–solving	25 (25–75)	Increase
	Difficulties with emotion regulation	Difficulties in Emotion Regulation Scale–18 (Kaufman et al., 2016)	1 item about self–isolation	19 (19–95)	Decrease
	Impact of self–defined problems	Psychological Outcome Profiles (PSYCLOPS; Sales et al., 2023)	None	4 (0–20)	Decrease
Adolescent	Emotional and behavioural difficulties (internalising, externalising and somatic symptoms; social and academic difficulties)	Adolescent–report Paediatric Symptom Checklist 17 (PSC–17; Brown et al., under review)	None	17 (0–34)	Decrease
		Caregiver report Paediatric Symptom Checklist 35 (PSC–35; Brown et al., under review)	None	35 (0–70)	Decrease
	Quality of life	Kid–KINDL (Bullinger et al., 2008)	None	24 (24–120)	Increase
Family	Family functioning	Adolescent– and caregiver–report Systemic Clinical Outcome and Routine Evaluation (SCORE; (Stratton et al., 2010)	1 item about satisfaction with family decision–making	16 (16–80)	Decrease

* Assessment items added through consultation with local study advisors.

### Trial feasibility and safety

#### Randomisation

Families were randomly allocated to Intervention or EUC using a 1:1 randomisation sequence which was computer-generated by an independent statistician using Research Randomizer (randomizer.org) with two blocks of 30, in order to allow staggered study arm allocation and intervention commencement. The statistician matched eligible family IDs to the allocation sequence and shared these back with the study coordinator on-site.

#### Masking

Assessors and principal investigators were masked to allocations of families, while implementing staff and participants were not masked. All staff were trained in the importance of maintaining masking. Prior to endline assessments, participants were instructed not to reveal their allocation to assessors. In cases where allocations were revealed, assessors were instructed to inform their coordinator immediately, who would assign another assessor to complete the assessment. To evaluate the level of (un)masking, assessors were asked to guess participant allocation after each endline assessment, including reasons for this guess.

#### Spill-over

To descriptively assess spill-over of intervention content to control participants, Intervention participants were asked at endline about the extent to which they shared information about the intervention with others, and EUC participants were asked whether they had heard about the intervention content from others.

#### Adverse events and referrals

We trained all study staff to monitor and report the occurrence of specific serious adverse events (SAEs) and adverse events (AEs) to the study coordinator, who then reported these to principal investigators, a Data Safety Management Committee (DSMC), and the ethical board. For urgent referral needs identified, study staff referred cases to a case management focal point who assessed and referred them as needed.

### Process evaluation

After endline we conducted 36 key informant interviews with implementation staff (*n* = 3; i.e., those coordinating the implementation of the intervention in the community centre) and caregivers (*n* = 19) and adolescents (*n* = 14) from 10 families who completed the intervention, 1 family who dropped out, and 5 EUC families. We conducted focus group discussions with facilitators (*n =* 4) and trainer/supervisor and master supervisor (*n =* 2). Assessors conducted the interviews using semi-structured guides exploring perceived acceptability, feasibility, and impact of the intervention, facilitators and barriers to implementation, and recommendations for improvements.

### Analysis

#### Quantitative analysis

Descriptive statistics (means, standard deviations, *N*’s, percentages) were used to explore baseline demographic characteristics. Cronbach’s alpha was used to evaluate the internal reliability of outcome measures at baseline.

To assess sensitivity to change of each outcome measure, we explored within-group change from baseline to endline for intervention and control groups through calculating means, standard deviations, within-group *t*-tests, and Cohen’s *d* effect sizes. In the case of missing item-level data, participant-level mean imputation was used. In the case of missing data on an entire outcome measure, the participant’s score was omitted from that time point, given that no regression models were conducted. Since this was a feasibility study, no between-group significance testing was conducted. Analyses were conducted using Stata15.

#### Qualitative analysis

Qualitative data were analysed using inductive and deductive thematic techniques (Braun and Clarke, 2006). After familiarisation with the data, a codebook was agreed and applied by two authors (AB and ACEB), grouped into five key topic areas with relevant sub-topics based on the interview guide and research questions. Three transcripts were double-coded to ensure consistency in coding and adequacy of the codebook, and the remainder were split between coders, with regular discussion to ensure consistency. Emerging themes were discussed and agreed during coding. Content within each sub-theme and theme were summarised, after which specific quotes were selected to illustrate them. These were individually reviewed for consistency and appropriateness and reviewed holistically to ensure applicability and comprehensiveness for the data set, with validation from the full study team and study advisors. No new ideas were identified during analysis of the final transcripts, suggesting saturation was achieved. Data was best represented by the following five themes (full findings are available on request): 1) Process and implementation factors important for impact; 2) Strong engagement and uptake; 3) Intervention content perceived as relevant and useful; 4) Perceived positive impacts within the family system; 5) Several perceived mechanisms of change. In line with our mixed-methods approach, qualitative findings are presented alongside quantitative and implementation data to answer the research questions.

## Results

### Sample characteristics

Table 3 provides demographic characteristics of adolescents (*n* = 104 adolescent-report; 126 caregiver-report), caregivers (*n* = 77), and families (*n* = 60). The majority of included caregivers were mothers (72%) and married (90%), with an average age of 42.5 years (range 29–59). Fifty-two percent of the sample were of Iraqi nationality, 40% Syrian, and 8% Jordanian. Most caregivers had secondary-level education or less, no caregivers were employed in full-time work, and average monthly household income was low at 220 JD (national minimum wage was 260 JD at the time of the study). The adolescent sample consisted of approximately equal numbers of males and females, with a good distribution of ages (*M =* 13.11, *SD =* 2.27), with the exception of few 16–17 year olds. Most adolescents (86%) attended school. There were no substantial demographic differences between intervention and control groups.

Hypothesis 1. Outreach, screening, attendance, and retention.

**Table 3.** Demographic characteristics of families in fRCT of Nurturing Families intervention

Family-level demographics, *N* (%)			
	Control *n =* 30	Intervention *n =* 30	Total *n =* 60
Monthly income (JD)			
Mean^ (SD) [Range]	232.16 (136.38)	207.76 (108.18)	219.96 (123.18)
	[0–500]	[0–410]	[0–500]
Type of home			
Not shared	26 (87%)	25 (84%)	51 (85%)
Shared	4 (13%)	5 (16%)	9 (15%)
Family composition			
Number of adults			
1	4 (13%)	1 (3%)	5 (8%)
2	16 (54%)	15 (50%)	31 (52%)
3–4	6 (20%)	10 (34%)	16 (27%)
5–7	4 (13%)	4 (13%)	8 (13%)
Number of children			
1	4 (13%)	3 (10%)	7 (12%)
2	6 (20%)	7 (23%)	13 (21%)
3–4	16 (54%)	17 (57%)	33 (55%)
5–7	4 (13%)	3 (10%)	7 (12%)

Caregiver demographics, *N* (%)			
	Control *n* = 37	Intervention *n =* 40	Total *n* = 77
Age	41.94 (6.96)	43 (7.42)	42.49 (7.17)
Mean (SD) [Range]	[30–54]	[29–59]	[29–59]
Caregiver type			
Father	10 (27%)	11 (27%)	21 (27%)
Mother	27 (73%)	29 (73%)	56 (73%)
Nationality			
Syrian	15 (40%)	16 (40%)	31 (40%)
Jordanian	5 (14%)	1 (3%)	6 (8%)
Iraqi	17 (46%)	23 (57%)	40 (52%)
Country of birth			
Jordan	3 (8%)	1 (2%)	4 (5%)
Syria	16 (43%)	16 (40%)	32 (41%)
Iraq	17 (45%)	23 (57%)	40 (51%)
Other	1 (2%)	0 (0%)	1 (1%)
Year arrived to Jordan*			
2005	1 (3%)	0 (0%)	1 (1%)
2011–2013	15 (44%)	15 (38%)	30 (42%)
2014–2016	10 (29%)	7 (18%)	17 (23%)
2017–2019	8 (24%)	15 (38%)	23 (32%)
2021	0 (0%)	2 (6%)	2 (2%)
Education			
None	0 (0%)	2 (5%)	2 (2%)
Elementary	21 (57%)	20 (50%)	41 (53%)
Secondary	9 (24%)	13 (33%)	22 (29%)
Vocational	2 (5%)	1 (2%)	3 (4%)
College/University	5 (14%)	4 (10%)	9 (12%)
Occupation			
Self–employed	4 (11%)	2 (5%)	6 (8%)
Daily wage	1 (3%)	1 (2%)	2 (3%)
Out of work – looking	4 (10%)	4 (10%)	8 (10%)
Out of work – not looking	2 (5%)	5 (13%)	7 (9%)
Homemaker	17 (46%)	21 (54%)	38 (49%)
Unable to work	0 (0%)	1 (2%)	1 (2%)
Volunteer	2 (5%)	1 (2%)	3 (3%)
Retired	0 (0%)	1 (2%)	1 (2%)
Not answered	7 (19%)	4 (10%)	11 (14%)
Marital status			
Married	33 (90%)	37 (93%)	70 (92%)
Divorced	2 (5%)	1 (2%)	3 (4%)
Widowed	0 (0%)	2 (5%)	2 (2%)
Separated	2 (5%)	0 (0%)	2 (2%)

Adolescent demographics, *N* (%)			
*n* (%)	Control *n =* 50	Intervention *n =* 54	Total *n =* 104
Age			
10–12 years	27 (54%)	21 (39%)	48 (46%)
13–15 years	12 (24%)	22 (41)	34 (33%)
16–17 years	11 (22%)	11 (20%)	22 (21%)
Gender			
Female	22 (44%)	29 (54%)	51 (49%)
Male	28 (56%)	25 (46%)	53 (51%)
Education			
Currently in school	52 (83%)	56 (89%)	108 (86%)
Previously in school	10 (16%)	7 (11%)	17 (13%)
Never in school	1 (1%)	0 (0%)	1 (1%)

* In the year arrived variable 4 families from Jordan are not counted.

^ JD = Jordanian Dinar; 1 JD approximately equal to 1.41 USD.

**Figure 2.**
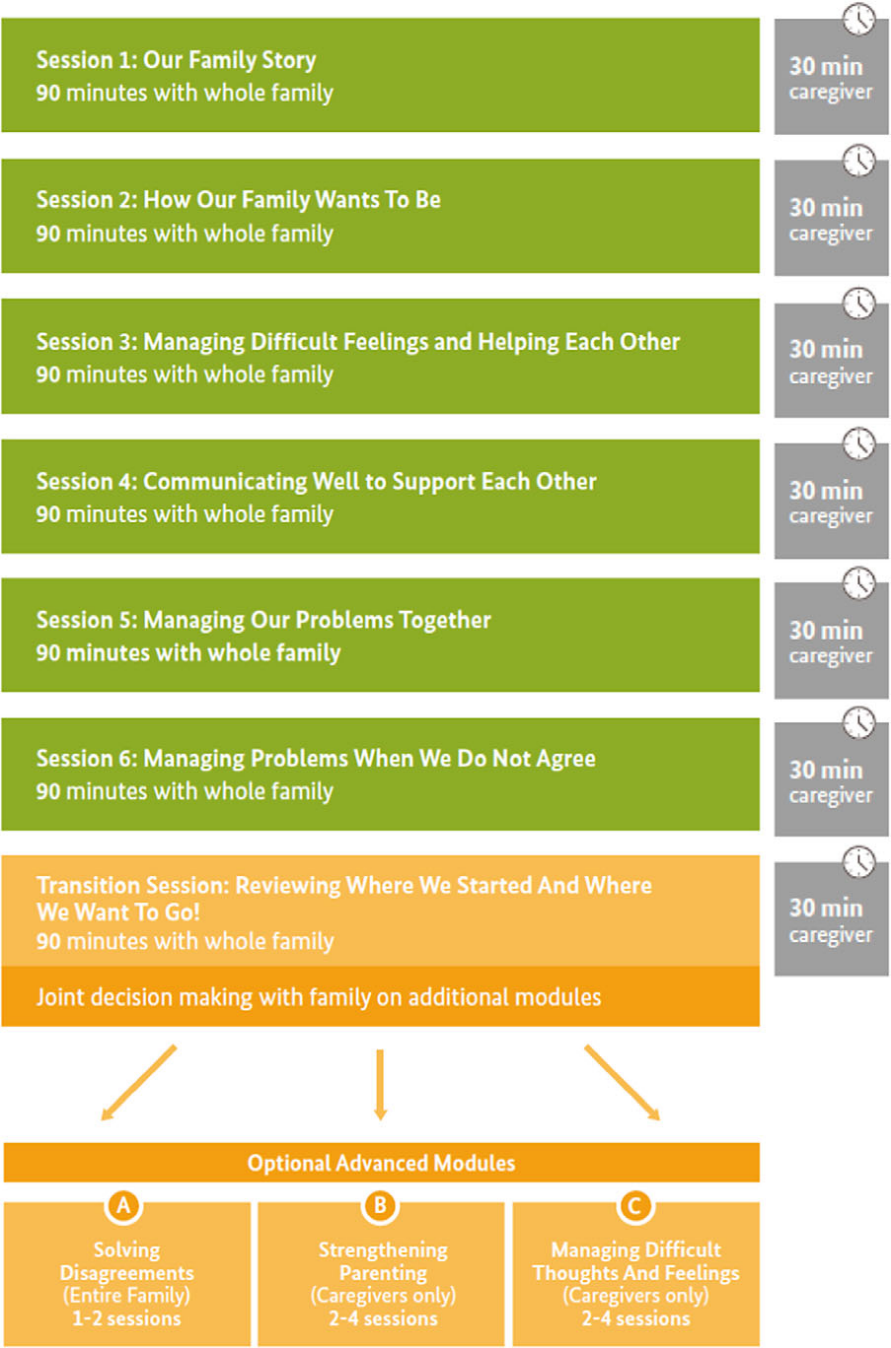
Consort flow chart for feasibility randomised controlled trial.

Within 3 weeks, 78 families were identified and contacted through outreach (see Figure 2). Seventy-seven of these families were invited for screening (one family was not invited, as maximum sample size was reached prior), and 62 completed screening (80%). Sixty-one families (98%) were eligible to take part, however, one declined, leaving a baseline sample of 60 families (77 caregivers, 55 mothers, 22 fathers; 104 adolescents, 53 males, 51 females). Based on screening, all families had at least one caregiver or adolescent scoring above the cut-off for distress. Family functioning challenges were reported by 77% of caregivers, and parenting challenges by 73%. Randomisation resulted in 30 families in each arm. At endline, 54 families (90%) completed assessments (61 caregivers, 76 adolescents).

#### Attendance in intervention sessions

Out of 30 allocated families, 27 participated in the intervention. One family then dropped out due to moving abroad. The remaining 26 families attended all six core sessions, and all except one (also due to relocating) attended the transition session. Of the 25 families remaining, 20 chose all three advanced modules (three completed two, one completed one, and one completed none).

#### Father attendance

In 56% of families, mothers attended the core module alone with adolescents (note that in 3 households, there was no father due to death [*n* = 1] or divorce [*n* = 2]). Of the 24 fathers in the sample, 8 attended all or most sessions (33%; with 1 father attending without the mother), 4 attended only some (17%), and 12 attended none (50%), primarily due to working (*n* = 4), lack of interest (*n* = 4), health-related issues (*n* = 2), other commitments (*n* = 1), or mother not wanting them to join (*n* = 1). Advanced modules were attended by fewer fathers; two fathers attended 6–7 sessions, five attended 3–4 sessions, and two attended 1–2 sessions.

#### Barriers and facilitators to uptake and engagement

Many caregivers reported having been open and curious to participate in the sessions, hoping to experience relief, reduce their *‘suffering’* and *‘pressures’,* and improve family communication and problem-solving. A few families admitted to having low expectations prior to starting the intervention, but that noticing early benefits encouraged ongoing engagement, with one family stating, *‘it was something beyond imagination’.* Several adolescents described being requested by their caregivers to attend and complying, without having much sense of what the intervention was. Some reported initial boredom, but increasingly enjoyed subsequent sessions. The main reported practical barriers to attendance included timing conflicts with schooling, exams, essential appointments and adolescent sleeping schedules, or travelling and health issues. Men were often unavailable due to work; given financial stress, livelihood opportunities took precedence over attending. However, fathers were also more likely to decline to attend based on preference alone. Implementing staff speculated that some more vulnerable or older adolescents may decline due to family conflict and hesitancy to speak openly with parents, and suggested special efforts are needed to reach these adolescents. Implementing staff described an intense effort required to schedule and reschedule sessions according to families’ needs, remind families of sessions, and follow up on non-attendance. Several interviewees recommended making more sessions available outside of standard working hours to reduce schedule conflicts, allow better engagement of fathers and older adolescents, and ease pressure on the number of rooms available.

#### Attendance and satisfaction with EUC financial literacy sessions

A total of 70 family members from 48 families (21 intervention, 27 control) attended the financial literacy sessions. Most family members (84.2% EUC, 78.1% Intervention) attended all three sessions, with similar rates between study arms. Qualitatively, participants were largely positive about the sessions and appreciated learning about financial planning, budgeting and saving. They found the content practical and applicable to their current and future financial situation. Some mentioned that communication in their household had improved – primarily about expense planning and saving methods.

#### Use of other services

Education supports were received by 3 EUC and 6 Intervention families, health services by 5 EUC and 2 Intervention families, and additional mental health services by 4 EUC and 1 Intervention family. No families reported receiving additional parenting, legal, or financial support. Additionally, as part of routine services and separate from the study, the community centre provided all families with a food voucher (85 JD) at the time of study completion.

Hypothesis 2. Feasibility, relevance, and acceptability of intervention.

#### Participant perceptions of intervention content, facilitators, and implementation

All participants stated that program content was culturally and contextually relevant, acceptable, and understandable. Strategies for problem management, emotion regulation, and communication were cited frequently as particularly powerful intervention strategies, and both adolescents and caregivers valued WhatsApp materials and reminders to support home practice. Most participants and facilitators described that the whole-family format, in a safe and supporting space, was beneficial for families to open-up, understand one another’s perspectives, and practice communication and problem-solving strategies using role plays and relevant examples. Despite finding it challenging at times to work with the whole family in session, facilitators highlighted the powerful impact they witnessed. Similarly, family members reported the value of bringing the family together:

The thing I liked the most… was when everyone used to share their opinions. It wasn’t that one would share their opinion and the other would say ‘it’s not nice and it’s not allowed’. I mean, each one had their opinion and respected other opinions. (Male, Iraqi, 12 years)

Some caregivers, adolescents, and implementing staff suggested having separate sessions for adolescents in addition to family sessions. In some cases, this referred to more general recreation opportunities, and in others, it referred specifically to additional content relevant to adolescent emotions:

The topics that we should focus on more?… Psychological problems, which are anxiety, worrying about the future… I mean, there is a lot of tension, anxiety. (Male, Iraqi, 16 years)

Generally, interviewees felt the content was more suitable for adolescents rather than younger children. A few participants requested more time to discuss their concerns in a less structured format. In terms of gender considerations, including gender-based violence, one adolescent girl suggested that content should include, ‘*material that raises parents’ awareness of early marriage, violence, or labour*’. Some mothers mentioned the particular relevance of the content to the reality of women, for example:

They had empathy. They hit the spot… we came from war…. we are renting houses. It is awful. We have zero money. The bigger problem is that the whole pressure is on the woman. The kids. Lack of money. The kids want this and that, they nag the woman. She is creating a volcano inside of her; anyone who wants to come near, “mother, mother”, I get angry at them…. They hit the spot and gave you solutions to that. They made you comfortable and they gave (help on) how to deal with this and that, how to prioritise your issues, all of these. (Mother, Syrian)

Participant feedback regarding facilitators was uniformly positive, and they were recognised as a powerful driver of intervention impact. Facilitators were perceived by caregivers and adolescents as non-judgemental and able to understand and empathise with participants’ situation (‘*one of us*’), and participants appreciated being listened to and accepted. Facilitators themselves reported extensive learning and personal and professional benefits from the experience, despite finding it emotionally challenging at times. Facilitators and other stakeholders cited quality training, close supervision, and strong staff-care and self-care for facilitators as essential to ensure the success of the intervention, with suggestions to further expand opportunities for individual professional development.

#### Facilitator fidelity and competency

According to facilitator-reported checklists, each session lasted on average 119 min (ranging 90–120 min; one outlier of 150 min) for core module sessions and 97 min (ranging 45–120 min) for advanced modules, in general alignment with the manual. Facilitators reported completing 100% of session components, indicating high fidelity. The trainer/supervisor observed 19 core sessions (10%) and 16 advanced modules (12%). Average supervisor-observed fidelity was 82% for core and 100% for advanced module sessions. Intervention-specific competency for session elements was mostly rated as ‘done well’ (79%) with only 13% ‘done partially well’ and 8% ‘needing improvement’.

Across the five core competency items, facilitators consistently used a range of basic skills in varying combinations. In many cases, facilitators were using *all* of the basic skills, and often showing advanced skills beyond what is commonly observed among beginning-level non-specialists. Importantly, there were very few instances of facilitators exhibiting behaviours that would be unhelpful; these were only observed in three sessions during which the facilitator did not directly acknowledge distress when it occurred during the session. Across the three family-specific competencies, again, most facilitators used a range of helping skills and very rarely engaged in any unhelpful behaviours.

Hypothesis 3. Outcome measures, perceived impacts, and trends over time.

Overall, there was very little missing data; on most measures <1% of items were missing. Assessors did not report any difficulties with administering the measures. Table 4 presents each outcome measure: internal consistency (Cronbach’s alpha), means and standard deviations at each timepoint, within-subject *t*-test results from baseline to endline for each group, and Cohen’s *d* effect size for each group. Internal consistency for most outcome measures was high (> 0.8). Only the child-report Paediatric Symptom Checklist (PSC-17) had an alpha lower than acceptable (0.61). The EUC group did not show a significant change in any outcome measure, while there were significant improvements from baseline to endline for many measures within the intervention group.

**Table 4.** Quantitative outcomes for Intervention and EUC groups from baseline to endline

Caregiver-reported outcomes								
	Enhanced Usual Care				Intervention			
Outcome and baseline alpha	Baseline (*N* = 37) M(SD)	Endline (*N* = 28) M(SD)	Within group *t*-test	*d*	Baseline (*N* = 40) M(SD)	Endline (*N* = 33) M(SD)	Within group *t*-test	*d*
PSC–35^a^ ^,^^b^ (α = 0.77)	28.25 (10.98)	28.55 (10.48)	*t*(50) = −0.48*, p* = .63	−0.02	28.27 (11.12)	20.79 (9.89)	***t*(52) = 5.14, *p* < .01**	0.58
Parenting (α = 0.88)	62.13 (7.28)	62.93 (7.36)	*t*(27) = −0.98, *p* = .33	−0.10	64.14 (6.81)	66.70 (4.00)	***t*(32) = −2.85, *p* < .01**	−0.39
K10^c^ (α = 0.85)	35.63 (8.21)	32.71 (9.53)	*t*(27) = 1.99, *p* = .06	0.29	34.90 (7.68)	23.00 (9.51)	***t*(32) = 8.13, *p* < .01**	1.21
SCORE (α = 0.89)	42.05 (10.79)	40.60 (11.17)	*t*(27) = −1.37, *p* = .18	0.11	39.64 (12.27)	33.67 (8.72)	***t*(32) = 5.03, *p* < .01**	0.48
DERS (α = 0.87)	58.19 (13.42)	56.38 (14.31)	*t*(27) = 0.57, *p* = .57	0.11	55.88 (15.82)	43.18 (13.18)	***t*(32) = 4.99, *p* < .01**	0.75
PSYCLOPS (α n/a)^d^	16.37 (3.53)	15.93 (3.09)	*t*(26) = 0.53, *p* = .60	0.11	16.19 (3.22)	12.97 (4.63)	***t*(30) = 4.41, *p* < .01**	0.72

Adolescent-reported outcomes								
	Enhanced Usual Care				Intervention			
Outcome and baseline alpha	Baseline (*N* = 50) M(SD)	Endline (*N* = 36) M(SD)	Within group *t*-test	*d*	Baseline (*N* = 54) M(SD)	Endline (*N* = 40) M(SD)	Within group *t*-test	*d*
PSC–17 (α = 0.61)	12.57 (4.45)	12.47 (5.06)	*t*(35) = 1.27, *p* = .21	0.02	12.84 (4.48)	11.98 (5.14)	*t*(39) = 1.55, *p.* 13	0.17
Kid–KINDL (α = 0.88)	85.67 (18.00)	84.53 (20.43)	*t*(35) = −0.10, *p* = .92	0.06	85.32 (15.64)	87.74 (15.17)	*t*(39) = −1.73, *p* = .09	−0.13
SCORE (α = 0.90)	36.61 (13.77)	38.56 (15.13)	*t*(35) = −0.65, *p* = .52	−0.12	35.49 (11.09)	33.35 (8.68)	***t*(39) = 2.84, *p* < .01**	0.17

Notes: M, Mean; SD, Standard deviation; d, Cohen’s d effect size; n, sample size. Bolded cells refer to significant findings.

Ms and SDs are raw means from all available data. Within group *t*-test is based on matched pair data from baseline to endline.

a *n* for PSC35 is Baseline treatment: 63; control: 63; Endline treatment: 53; control: 51.

b Dropped for one family where different caregivers completed at baseline versus endline.

c 1 caregiver did only K10 at Baseline, so K10 *n* = 78 (38 control, 40 intervention), while remainder *n* = 77.

d *Endline PSYCLOPS only has 58 entries, not collected for 2 caregivers in intervention group and 1 in control.*

#### Caregiver outcomes

Significant improvements were demonstrated in caregiver distress (*d* = 1.21, *p* < .01), parenting practices (*d* = 0.39, *p* < .01), emotional regulation (*d* = 0.75, *p* < .01), and impact of self-defined problems (*d* = 0.72; *p* < .01). Caregivers, adolescents, and facilitators spoke of qualitative improvements to caregiver mood and caregivers often mentioned having a greater ability to curb their anger or irritation, and as a result were able respond to daily stressors – particularly those relating to their children – in a less reactive and more considered way that better reflected their parenting values.

#### Adolescent outcomes

Findings for adolescent outcomes were mixed. Qualitatively, caregivers reported improvements in adolescent well-being, while adolescents were less likely to describe improvements to their own mood. This corresponds with quantitative data whereby we saw caregiver-reported improvements on the PSC-35 (*d* = 0.50; *p* < .01), but no significant adolescent-reported improvements in psychological distress (PSC-17) or well-being (Kid-KINDL).

#### Family-level outcomes

Quantitatively, both adolescents (*d* = 0.17, *p* < .01) and caregivers (*d* = 0.48, *p* < .01) reported improved family functioning, and this was triangulated with qualitative data. Caregivers commonly reported increased cooperation and helpfulness from adolescents, and some adolescents reported having improved their behaviour towards others, listening better, showing others more respect and interacting more positively and empathetically with caregivers, siblings and peers. Caregivers, adolescents, and facilitators consistently spoke of improvements in family communication and explicitly highlighted this as a mechanism of positive impacts on individuals. Interviewees reported improved bonding, sharing more positive moments, appreciating one another more, caregivers discussing adolescent concerns, and adolescents increasingly trusting their caregivers. This change in family communication and dynamic was the most prominent impact described, in strong accord with the aim of the intervention to increase communication and positive family interactions in order to improve caregiver mental health and subsequently child well-being. One mother describes:

There was distance among the family members. Now, I express to them that I love them… I hug my children… I sit with them more, I understand them. If I saw one of them annoyed, I ask what’s wrong… I was the one who changed. I felt that everything changed when I changed myself. (Mother, Syrian)

Hypothesis 4. Safety and feasibility of trial procedures.

#### Randomisation

Randomisation resulted in approximately equal group sizes, with no notable demographic differences between groups. Interventions were delivered as allocated. Some families from the EUC group expressed disappointment at not receiving the Intervention, as they had heard about it from neighbours and friends.

#### Masking

Assessors correctly guessed 83% of allocations. These findings indicate unmasking during many assessments. Assessors reported that family members disclosed details about their allocation on three occasions. Assessors informed the research coordinator about disclosures, however, assigning a new assessor to complete the remainder of the assessment was often not possible.

#### Spill-over

At endline, only one caregiver in the control group reported having received general information about the intervention from a friend. However, in the intervention group, 25 caregivers reported that they had shared key learning points from the intervention with others, including friends, neighbours, and extended family. This indicates the potential for spill-over to be of concern in future studies.

#### Adverse *events and referrals*

Five adverse events were reported: four child protection concerns, and one case of emotional abuse against a caregiver. All were reported to case management and the DSMC, who judged these events as not linked to the study or intervention and were satisfied with follow-up measures taken. No serious adverse events or child safeguarding concerns were reported. Additional referrals were needed for 11 families (five financial, two educational, two mental health, one behavioural therapy and one physiotherapy), and actioned.

## Discussion

The aim of this fRCT of the Nurturing Families intervention in Jordan was to assess the feasibility, relevance, safety, and acceptability of the whole-family intervention and research protocols in preparation for a fully-powered RCT. Overall, findings indicated that delivery of the intervention by non-specialist facilitators was safe, feasible, acceptable and relevant for families. Facilitators were able to deliver the content with adequate fidelity and competency, with high-quality training, and regular supportive supervision cited as essential to ensure quality. To support implementation of this and other task-sharing approaches at scale, high-quality implementation science research should explore the optimal ways to train, supervise, and monitor for sustained quality (Tol et al., 2023).

There was high intervention interest, uptake, and retention by families, with most drop-out due to practical reasons rather than dissatisfaction. Challenges with uptake of psychological interventions have been noted in various studies in the region (Pluess et al., 2022; Brown et al., 2023) and based on qualitative findings, comparative success in this project may have been due to the family-system focus (which fit the perceived needs of families), high relevance of intervention content (with strategies of communication, problem solving, and emotion regulation cited as most powerful), and outreach being conducted by trusted members of the community. Other family-focused interventions have shown similar promise in LMIC contexts (Betancourt et al., 2020), and our findings show the relevance for settings with high rates of forced displacement. Broadening our conceptualisation and treatment of mental health and well-being in contexts of adversity to collective mental health paradigms and focusing on interpersonal processes at family and community levels may improve cultural and contextual fit and ultimately enhance reach and impact (Bosqui, 2020).

Research procedures were largely found to be feasible and acceptable, but careful planning will be needed in future studies to maintain assessor masking, and alternative study designs should be considered to prevent potential spill-over of intervention content (such as cluster-RCTs), and discontent around not receiving intervention (such as using a wait-list). In this study, the lack of masking at endline is a limitation since outcomes may have been unintentionally biased. Our outreach method using the adapted ReachNow tool (van den Broek et al., 2023) yielded a high accuracy rate, with 98% of screened families being eligible. Since other research similarly shows consistently accurate levels of detection and improved help-seeking behaviour (Jordans et al., 2015), this tool should therefore be considered for future implementation to facilitate low-cost, non-stigmatising methods to identify families in need of support. The multi-dimensional screening interview could be feasibly implemented by trained assessors and can be considered for future use in place of costly clinician assessments and triage. Future research should be designed in such a way that allows measurement of the specific contributions of each intervention module, in order to improve targeting and better understand active ingredients.

Although not powered to assess between-group effects, findings show promising indications of the effects of the intervention. EUC group families did not improve significantly on any outcome, while intervention group families improved on all caregiver-reported outcomes, with moderate to large effect sizes. Adolescent-reported outcomes were more varied, with some qualitative reports of improvements, but quantitative data showed only small significant improvements in family functioning in the intervention group and no changes in adolescent-reported distress and well-being. This contrasts with the caregiver-reported measure of adolescent distress, which showed significant, medium-size changes. It must be noted that the PSC-17 measure completed by adolescents showed below-adequate internal consistency. Additionally, the baseline mean score was 12, which represents the clinical cut-off for this measure (Brown et al., under review), indicating that average adolescent-reported levels of distress in this sample at baseline were not particularly elevated.

Future research should more carefully consider how to measure adolescent outcomes on the one hand, but also consider linking to additional psychosocial support opportunities for adolescents or building in an adolescent-only module for the intervention to enhance outcomes for adolescents. Given the known impact of parenting and family processes on child and adolescent mental health and well-being (Eltanamly et al., 2021) and the potential of parenting and family programmes to effect changes in child and adolescent outcomes (Pedersen et al., 2019), in line with our assumptions it is also possible that the large intervention effects on parenting practices, caregiver mental health, and family functioning may lead to later improvements in adolescent outcomes. Follow-up assessments should be included in future studies to assess this. Fully-powered studies should also include analysis of sub-scale scores of measures to pinpoint more specific changes occurring in family relationships, caregiving practices, and adolescent and caregiver mental health and well-being.

Several challenges were noted that may impact effectiveness of the intervention at scale. In our sample, 50% of fathers engaged in some sessions, and 33% engaged in most or all. Although this represents higher levels of father engagement than have been found in parenting interventions globally (Panter-Brick et al., 2014), more focused attention to the best ways to support father attendance is needed. Outreach by trusted community members, flexibility in scheduling sessions around work commitments, and methods to share content in the case of non-attendance were found to be key in our study and may have helped overcome perceptions that such interventions were targeted only at mothers. Relatedly, efforts should be made to strengthen the ways in which gender dynamics, and family violence are specifically addressed in the intervention - either through bolstering content explicitly addressing harmful gender norms and/or learning how to adjust the intervention in cases where violence is present, given potential harms of whole-family approaches in these cases. Our approach for the fRCT was to refer to case management when issues including violence and other protection risks arose, however, this may have limited feasibility and sustainability at scale, particularly in settings where quality protection services that work for the best interests of women and children are lacking or overburdened.

Similarly, there were high levels of financial need within our sample, and while the psychosocial support was reported to be beneficial, we had repeated requests for financial support and higher transportation reimbursements. The potential for ongoing adversity to limit intervention impact has been reported in numerous process evaluations of psychological interventions in humanitarian settings (e.g., Miller et al., 2022; Brown et al., 2023) and highlights the need for holistic, integrated, multi-sector responses (Weissbecker et al., 2023). While this is likely to require structural change to overcome siloed approaches to humanitarian responses, our findings indicated the financial literacy course offered as part of EUC was well received, with high attendance and positive feedback. Given the pervasive financial challenges faced by families, work is underway to integrate a financial literacy module into the NF intervention. Future research will be needed to test the additive benefits of such a module, alongside links to other sectors, to more comprehensively ensure basic needs are met.

## Conclusion

Findings from this feasibility RCT indicate that the NF family-systemic intervention and study procedures are overall safe, feasible, acceptable, and highly relevant for urban refugee and host community families in Jordan, and there were promising improvements in caregiver and family outcomes after receiving the intervention. The whole-family format, practical skills-based focus, and local facilitators supported through high-quality training and supervision were perceived as important ingredients for success. Challenges to address in future research and implementation include improving engagement and impact for adolescents, understanding how to optimise father engagement, and improving mechanisms for managing gender-related issues, family violence, and responding to poverty and other pervasive social determinants of mental health in families. Future research should be carefully designed to maintain assessor masking and avoid spill-over effects. After some adaptation to overcome these challenges, we believe that the criteria have been met for progression to a fully powered RCT to evaluate effectiveness.

## Supporting information

Brown et al. supplementary material Brown et al. supplementary material

## Data Availability

The data that support the findings of this study are available from Mark Jordans (mark.jordans@warchild.nl) upon reasonable request.

## Supplementary material

The supplementary material for this article can be found at http://doi.org/10.1017/gmh.2024.43.
