# Nurturing families: A feasibility randomised controlled trial of a whole-family intervention with vulnerable families in Jordan – ERRATUM

**DOI:** 10.1017/gmh.2024.64

**Published:** 2024-06-04

**Authors:** Felicity L. Brown, Hind Yousef, Alexandra C.E. Bleile, Hadeel Mansour, Anna Barrett, Maha Ghatasheh, Eve S. Puffer, Zeinab Mansour, Karam Hayef, Samer Kurdi, Qaasim Ali, Wietse A. Tol, Aala El-Khani, Rachel Calam, Hana Abu Hassan, Mark J.D. Jordans

The publisher apologises that upon publication the images for figure 1 and 2 were transposed causing the images to be shown and listed in the wrong order.

Additionally an extra ‘and’ was included in the corresponding author information.

These have been corrected in the article
