# Supplementary material for: Nurturing families: A feasibility randomised controlled trial of a whole-family intervention with vulnerable families in Jordan
Source: Glob Ment Health (Camb). 2024 Apr 11;11:e51. doi: 10.1017/gmh.2024.43 (PMC11076925; doi:10.1017/gmh.2024.43)
Supplement: Peer review history

# Editor report: Recommendation: Nurturing families: A feasibility randomised controlled trial of a whole-family intervention with vulnerable families in Jordan — R0/PR2

**DOI:** 10.1017/gmh.2024.43.pr2

**Affiliations:** Makerere University College of Health Sciences, Uganda

Dear authors,

please find attached revise the manuscripts as per the reviewers comments and ensure to follow with the manuscript guidelines. ** Insert Table 1 about here** same to figures. We recommend that you cite the figure or table in the text. Where the table/figure has been cited, the table or figure will be inserted after acceptance of the manuscript

# Editor report: Decision: Nurturing families: A feasibility randomised controlled trial of a whole-family intervention with vulnerable families in Jordan — R0/PR3

**DOI:** 10.1017/gmh.2024.43.pr3

**Affiliations:** Johns Hopkins University Bloomberg School of Public Health, United States

No accompanying comment.

# Editor report: Recommendation: Nurturing families: A feasibility randomised controlled trial of a whole-family intervention with vulnerable families in Jordan — R1/PR5

**DOI:** 10.1017/gmh.2024.43.pr5

**Affiliations:** Makerere University College of Health Sciences, Uganda

This manuscript is provisionally accepted pending authors revision to minor comments raise by the reviewers on the revised version of the manuscript.

# Editor report: Decision: Nurturing families: A feasibility randomised controlled trial of a whole-family intervention with vulnerable families in Jordan — R1/PR6

**DOI:** 10.1017/gmh.2024.43.pr6

**Affiliations:** Johns Hopkins University Bloomberg School of Public Health, United States

No accompanying comment.

# Editor report: Recommendation: Nurturing families: A feasibility randomised controlled trial of a whole-family intervention with vulnerable families in Jordan — R2/PR8

**DOI:** 10.1017/gmh.2024.43.pr8

**Affiliations:** Makerere University College of Health Sciences, Uganda

After careful review of the responses to reviewers comments. The manuscript is accepted for publication.

# Editor report: Decision: Nurturing families: A feasibility randomised controlled trial of a whole-family intervention with vulnerable families in Jordan — R2/PR9

**DOI:** 10.1017/gmh.2024.43.pr9

**Affiliations:** Johns Hopkins University Bloomberg School of Public Health, United States

No accompanying comment.
